# Heterogeneous Distribution of Erucic Acid in *Brassica napus* Seeds

**DOI:** 10.3389/fpls.2019.01744

**Published:** 2020-01-29

**Authors:** Shaoping Lu, Mina Aziz, Drew Sturtevant, Kent D. Chapman, Liang Guo

**Affiliations:** ^1^ National Key Laboratory of Crop Genetic Improvement, Huazhong Agricultural University, Wuhan, China; ^2^ Center for Plant Lipid Research and Department of Biological Sciences, University of North Texas, Denton, TX, United States; ^3^ BioDiscovery Institute, University of North Texas, Denton, TX, United States; ^4^ University of Texas Southwestern Medical Center, Dallas, TX, United States

**Keywords:** *Brassica napus*, canola, erucic acid, spatial distribution, matrix assisted laser desorption/ionization-mass spectrometry imaging

## Abstract

*Brassica napus* (*B. napus*) is the world's most widely grown temperate oilseed crop. Although breeding for human consumption has led to removal of erucic acid from refined canola oils, there is renewed interest in the industrial uses of erucic acid derived from *B. napus*, and there is a rich germplasm available for use. Here, low- and high-erucic acid accessions of *B. napus* seeds were examined for the distribution of erucic acid-containing lipids and the gene transcripts encoding the enzymes involved in pathways for its incorporation into triacylglycerols (TAGs) across the major tissues of the seeds. In general, the results indicate that a heterogeneous distribution of erucic acid across *B. napus* seed tissues was contributed by two isoforms (out of six) of *FATTY ACYL COA ELONGASE* (*FAE1)* and a combination of phospholipid:diacylglycerol acyltransferase (PDAT)- and diacylglycerol acyltransferase (DGAT)-mediated incorporation of erucic acid into TAGs in cotyledonary tissues. An absence of the expression of these two *FAE1* isoforms accounted for the absence of erucic acid in the TAGs of the low-erucic accession.

## Introduction

*Brassica napus* (AACC, 2n = 38) is an allotetraploid oilseed plant species formed by the hybridization of two diploid species of *Brassica rapa* (AA, 2n = 20) and *Brassica oleracea* (CC, 2n = 18) about 7,500 years ago (Chalhoub et al., 2014; An et al., 2019). It is the third largest oil crop in the world and accounts for approximately 15% of the vegetable oil used for human consumption (Wells et al., 2014; Liu et al., 2016; Carruthers et al., 2017; Kaur et al., 2019). Like most oilseeds, triacylglycerols (TAGs) comprise 95% of *B. napus* seed oil which are composed of a glycerol backbone esterified with three fatty acyl chains (Ai et al., 2014; Guan et al., 2016). These fatty acids vary in levels of saturation and carbon lengths and can contain mono/polyunsaturated fatty acids such as oleic (C18:1), linoleic (C18:2), linolenic (C18:3) and erucic (C22:1) acids, and/or saturated fatty acids such as palmitic acid (C16:0) and stearic acid (C18:0) (Zhao et al., 2007; Lu et al., 2016; Zhao et al., 2019).

There are two major seed-oil types of *B. napus*, low-erucic acid type and high-erucic acid type. Low erucic acid content (< 2%) is a major evaluation index for edible rapeseed cultivars (Hristov et al., 2011; Yan et al., 2015), and reducing erucic acid content has been a major goal for rapeseed breeding programs (Yan et al., 2015; Zhao et al., 2019). Alternatively, cultivars of *B. napus* containing high erucic acid are an important resource for industrial applications (Hristov et al., 2011). Recently, with the increasing demand for biodegradable and environmentally safe oil products such as biodiesel, lubricants, surfactants, pharmaceuticals, cosmetics, soaps, rubber and nylon, there has been renewed demand for erucic acid from high-erucic acid rapeseed (Hristov et al., 2011; Li et al., 2012; Konkol et al., 2019). In fact, *B. napus* cultivars were naturally high in erucic acid before the canola cultivar was bred for human consumption in 1974 (Hristov et al., 2011).

Although *de novo* fatty acid synthesis occurs in the plastids, long-chain monounsaturated fatty acids are formed in the cytoplasm by a membrane-bound FATTY ACYL COA ELONGASE (FAE) complex on the endoplasmic reticulum (ER) (Katavic et al., 2002). The plastid-produced oleic acid (C18:1) is the initial substrate for the FAE complex to generate erucic acid (C22:1) in *B. napus*, through two cycles of elongation. Each round of elongation involves four reactions catalyzed by the FAE complex. The first step involves a condensation of the C18:1-CoA with malonyl-CoA to generate the corresponding 3-ketoacyl-CoA. This 3-ketoacyl-CoA is then reduced to a 3-hydroxyacyl-CoA derivative that undergoes sequential dehydration and reduction to generate the elongated acyl-CoA final product (Katavic et al., 2002). FAE1 is the condensing enzyme that catalyzes the first of four reactions of the FAE complex, and is the rate-limiting enzyme that controls erucic acid accumulation in *B. napus* (Millar and Kunst, 1997). In *B. napus*, there are six paralogs encoding FAE1 proteins (Qiu et al., 2006; Wu et al., 2008; Cao et al., 2010). *BnaA8.FAE1* and *BnaC3.FAE1* are the two major genes responsible for erucic acid synthesis in *B. napus* seeds and they elongate 18:1-CoA to 20:1-CoA, and then 20:1-CoA to 22:1-CoA (Furmanek et al., 2014; Kaur et al., 2019). These two genes are highly expressed in the seeds of high-erucic acid varieties and are minimally expressed in low- erucic acid varieties (Qiu et al., 2006; Cao et al., 2010). After the formation of 22:1-CoA, it can be acylated to the glycerol backbone by enzymes in either the canonical Kennedy or Lands pathways to ultimately form TAG (Chapman and Ohlrogge, 2012; Furmanek et al., 2014).

In oilseeds, TAGs are primarily stored in the embryo, which consists of outer cotyledons (OC), inner cotyledons (IC), and an embryonic axis (EA) (Borisjuk et al., 2013; Woodfield et al., 2017; Lu et al., 2018). Previous studies of *B. napus* seeds with low erucic acid content have shown that total lipids and lipid molecular species are differentially distributed across its seed tissues (Borisjuk et al., 2013; Woodfield et al., 2017; Lu et al., 2018). Although the metabolism of erucic acid is understood, the spatial distribution of lipids containing erucic acid has not been explored. Matrix assisted laser desorption/ionization-mass spectrometry imaging (MALDI-MSI) is a mass spectrometry visualization platform for imaging metabolites *in situ* and has been an important tool for mapping the spatial distributions of glycerolipids in oilseeds, especially phosphatidylcholine (PC) and TAG (Horn and Chapman, 2014a; Sturtevant et al., 2015). Currently, MADLI-MSI has been used to analyze the spatial distribution of lipid metabolites in many oilseeds including cotton, castor, *Camelina*, *Arabidopsis* and low-erucic varieties of *B. napus* seeds (Horn et al., 2012; Horn et al., 2013; Horn et al., 2014; Horn and Chapman, 2014a; Sturtevant et al., 2016; Woodfield et al., 2017; Lu et al., 2018; Sturtevant et al., 2019). Here, two *B. napus* accessions, WH3401 (high-erucic) and WY20 (low-erucic), were comprehensively analyzed to compare their lipid distributions as well as gene expression profiles of *FAE1* and other related lipid biosynthesis genes. Results presented here will help elucidate the mechanisms for controlling the heterogeneous deposition of erucic acid in *B. napus* seed tissues.

## Materials and Methods

### Plant Seed Collection and Analysis

*B. napus* accessions, WH3401 and WY20, are part of a collection of natural and breeder-developed accessions that have been planted in Wuhan, China for many years. The agronomic traits of both accessions are stable. Mature seeds of field-grown plants were collected to measure oil content, using near infrared spectroscopy, and to determine erucic acid content. Seed weight (1,000-seed weight) and seed diameter were also measured. For developing seeds, flowers were labelled after bud opening and were bagged for seed selfing. Developing seeds were collected from 5–6 individual plants grown in the field on the campus of Huazhong Agricultural University. Seeds were collected from siliques 18, 23, 28, 33, 38, 43, 48, and 53 days after flowering (DAF), and were flash-frozen in liquid nitrogen for the analysis of seed dry weight, fatty acid composition, and TAG content, as well as for RNA extractions. Mature desiccated seeds were used for gas chromatography-flame ionization detector (GC-FID), MALDI-MSI and electrospray ionization-mass spectrometry (ESI-MS) analysis.

Mature desiccated seeds from 5-6 individual plants were dissected, and OC, IC, EA, and SC were separated under stereoscopic microscope and weighed as described previously (Lu et al., 2018). Fatty acid composition of dissected seed tissues, developing and mature seeds was quantified as methyl esters using a GC-FID (based on the internal standard heptadecanoic acid, C17:0, added at the time of extraction), following the method described by Lu et al. (2016).

### Tissue Preparation and Lipid Distribution Analysis by MALDI-MS Imaging

Mature desiccated seeds of two accessions were embedded in a 10% gelatin solution, frozen and cryo-sectioned as described previously (Sturtevant et al., 2015). Tissue sections were coated with 2, 5-dihydroxybenzoic acid (DHB; 98%, Sigma-Aldrich) by sublimation, following the method adapted from Hankin et al. (2007). Coated seed sections were analyzed by a hybrid MALDI-linear ion trap-Orbitrap mass spectrometer (MALDI-LTQ-Orbitrap XL; Thermo Scientific, San Jose, CA, USA) as described by Lu et al. (2018). MALDI-MSI data analysis and images processing were performed according to the method described by Horn and Chapman (2014b).

### ESI-MS Analysis of TAG and PC of Whole Seeds

Lipids were extracted from mature seeds as described by Chapman and Moore (1993) using hot-isopropanol to inactivate phospholipases. TAG (tri-17:0) and PC (di-14:0) (Sigma-Aldrich) were added into the extraction solution as internal standards. Crude lipid extracts were purified and neutral and polar lipids were separated and eluted as described previously (Lu et al., 2018). The neutral and polar lipid fractions were analyzed on an API 3000 mass spectrometer (SCIEX, https://sciex.com) to determine TAG and PC species and content. Instrument conditions were set as described by Welti et al. (2002) and Li et al. (2014). The molecular compositions of TAG and PC were determined from full MS scans and precursor ion fragment of the head group at *m/z* of 184.07, respectively. Data analysis used an open source software, LipidomeDB Data as described by Zhou et al. (2011).

### RNA Extraction and Real-Time PCR

Developing seeds collected from 3 individual plants were used for RNA extraction for real-time qPCR analysis. The RNA was extracted from 18, 23, 28, 33, 38, 43, 48, 53 DAF seeds and OC, IC, EA of 43 DAF seeds using RNAprep pure plant kit (DP432, http://www.tiangen.com/). The RNA extracts were used to synthesize the first-strand cDNA using an EasyScript RT Kit (AE311-03). Quantitative PCR (qPCR) was done using the BIO-RAD CFX96 qPCR detection system (Bio-Rad, http://www.bio-rad.com) with SYBR green to monitor dsDNA accumulation. The primers of *FAE1s* were designed to test the total expression of *FAE1b* and *f*, as well as *FAE1a* and *e* because they have highly similar sequences, respectively. All primers used for qPCR were listed in **Table S1**. The qPCR conditions were the same as described previously (Lu et al., 2018). Gene expression levels estimated by real-time qPCR were normalized to the levels of *BnACTIN*.

## Results and Discussion

### Comparative Analysis of Two *B. napus* Seeds Differing in Erucic Acid Content

**Figure 1:**
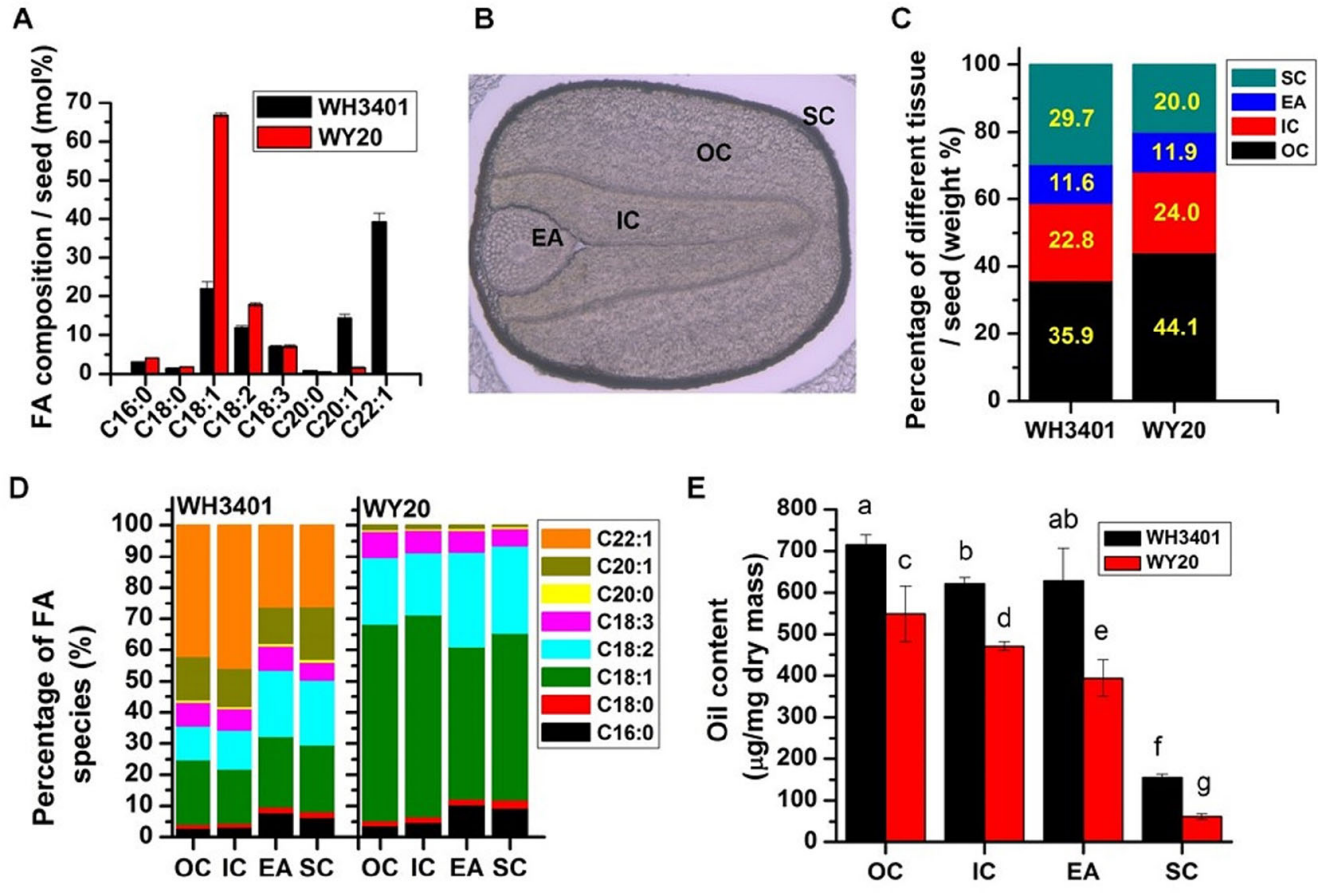
Oil content and fatty acid composition in dissected seed tissues. **(A)** Fatty acid composition in whole seeds (means ± SD, n = 6). **(B)** Seed structure under bright-field microscope. **(C)** Percentage of different tissues in whole seeds. **(D)** Fatty acid composition of different seed tissues. **(E)** Oil content of different seed tissues (means ± SD, n = 5–6). Letters denote significant difference at *P* < 0.05 using ANOVA analysis. FA, fatty acid; EA, embryonic axis; IC, inner cotyledon; OC, outer cotyledon; SC, seed coat.

In the current study, two natural *B. napus* accessions that differ in their erucic acid content were selected, WH3401 (high-erucic), which contains ca. 34% erucic acid, and WY20 (low-erucic) with almost no erucic acid content. The two accessions had different general and storage characteristics as summarized in **Table 1**. Compared to WH3401, WY20 had larger seed size and weight, but lower seed oil content. The seed oil content was measured by near-infrared spectrometry, and the average seed oil content over a three-year period was 54 and 42% in WH3401 and WY20, respectively (**Table 1**). Moreover, the fatty acid (FA) composition of the seeds was determined by GC-FID, and the two accessions exhibited different fatty acid profiles. The C18:1, C20:1, and C22:1 species constituted ca. 20, 15, and 40%, respectively, of the total fatty acid pool in the high-erucic accession. While in the low-erucic seeds, C18:1 was the major fatty acid, representing ca. 70% of the total fatty acid content, with only traces of C20:1 and C22:1 detected. The levels of the other fatty acids were similar between the two accessions (**Figure 1A**).

**Table 1:** The characteristics of high- and low-erucic acid rapeseed (*Brassica napus* L.).

Name	Seed oil content (%)	Erucic acid content (%)	Weight /1,000 seed (g)	Diameter (mm)
WH3401	54.52 ± 2.00	34.05 ± 0.26	3.31 ± 0.10	1.84 ± 0.04
WY20	42.60 ± 0.97**	0.20 ± 0.25**	4.00 ± 0.07**	1.99 ± 0.06**

Two inbreed accessions were naturalized in Hubei for many years, the agronomic traits trend to be stable. The data of seed oil content, erucic acid content and seed weight are the average value of 3 years. Open-pollen seeds were analyzed by near infrared spectrometer and 5 plants were analyzed for each accession every year. ** denotes significant difference at *P* < 0.01 using student *t*-test.

The different seed tissues of the two accessions, including the outer cotyledon (OC), inner cotyledon (IC), embryonic axis (EA), and seed coat (SC) were dissected, and separately analyzed for weight, fatty acid composition, and oil content (**Figure 1**); a representative image of the different seed tissues under bright-field microscope is shown (**Figure 1B**). The weight percentage of each of the four tissues per seed was similar between the two accessions with the OC having the highest proportion among the different tissues (**Figure 1C**). However, it seems that WH3401 has a relatively thicker seed coat, where the SC represents 30% of the seed weight, versus 20% in WY20 (**Figure 1C**). This difference in the seed coat was associated with a decrease in the proportion of the OC in WH3401 (35%) compared to WY20 (44%) (**Figure 1C**). Not surprisingly, the fatty acid composition in the four seed tissues does reflect that of the whole seeds for both accessions (**Figure 1D**). In WH3401, the C22:1 had the highest percentage (ca. 45%) in the outer and inner cotyledons, while in EA and SC tissues, C18:1, C18:2, and C22:1 nearly had equal proportions (ca. 25% each) of the total fatty acids. By contrast, in WY20, the C18:1 species was the most abundant in all the four seed tissues, it was ca. 62, 64, 48, and 53% in OC, IC, EA, and SC (**Figure 1D**). As with the whole-seed oil content, each of the dissected seed tissues had higher oil content in WH3401, relative to WY20, with the SC has the lowest oil content among all tissues in both accessions (**Figure 1E**).

**Figure 2:**
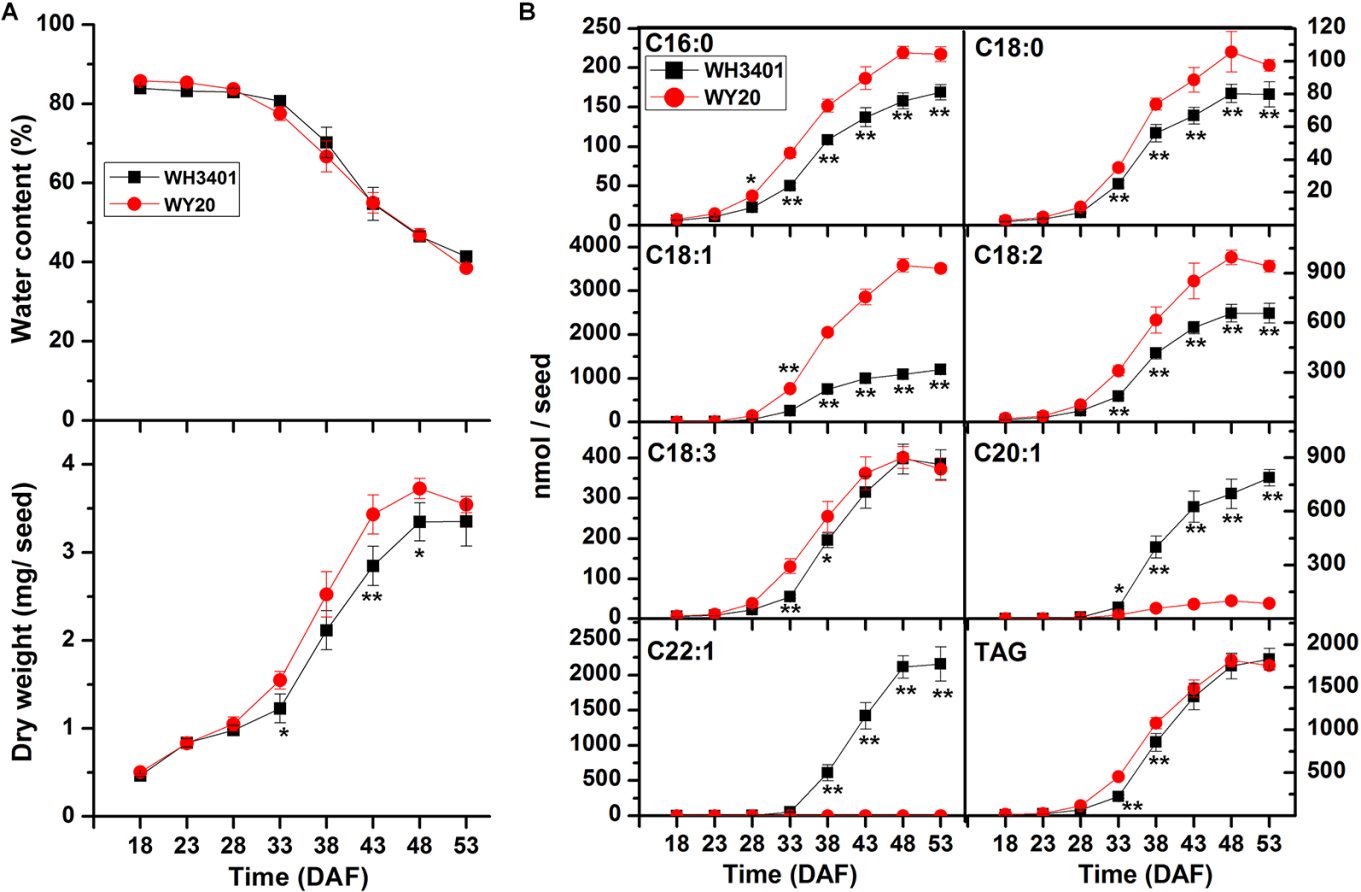
Characterization of seed development of WH3401 and WY20. **(A)** Water content and seed weight (dry weight) during seed development. Data of seed water content and seed weight are the means ± SD of 5 individual plants. **(B)** Fatty acid accumulation during seed development. Data of fatty acid content are the means ± SD of 6 individual plants. DAF, days after flowering. * and ** denote significant difference at *P* < 0.05 and *P* < 0.01, respectively, based on Student's *t* test.

Furthermore, water content, dry weight and FA accumulation were measured at different time points during seed development, spanning 18-53 DAF (**Figure 2**). Water content and dry weight mass were comparable between WH3401 and WY20 throughout seed development (**Figure 2A**). For FA accumulation, a difference was observed between the two accessions as early as 33 DAF, but it became more pronounced at later stages (**Figure 2B**). As expected, the accumulation of C20:1 and C22:1 fatty acid was greater in WH3401 than WY20, while opposite pattern was observed for the other fatty acids, especially the C16:0, C18:0, C18:1, and C18:2 species (**Figure 2B**).

### Spatial Distribution of TAG and PC in *B. napus* Seeds *in situ*

To get insights into the tissue distribution of TAGs and the metabolic precursors, PCs, seeds of both accessions were cryo-sectioned and analyzed with MALDI-MSI (Horn and Chapman, 2014a; Horn and Chapman, 2014b; Sturtevant et al., 2015; Woodfield et al., 2017) (**Figures. S1**, **S2** and **3**). High-resolution mass spectra were collected at each location on the tissue sections at 40-micron step size, and the data were analyzed by Metabolite Imager software (Horn and Chapman, 2014b). The ion intensities for TAG and PC molecular species were converted to mol%, and then plotted as false‐color images on a green (minimum) to red (maximum) scale representing the ion intensity corresponding to each *m/z,* with the scale adjusted individually to visualize the distribution of each molecular species across the seeds. Moreover, the relative levels of TAG and PC molecular species analyzed by MALDI-MSI (calculated as mol% from the ion intensities summed over the entire tissue section) were compared to those determined quantitatively in whole-seed extracts by ESI-MS. Both methods showed consistency in the measured average mol% for most of the determined molecular species (**Figure 4**).

**Figure 3:**
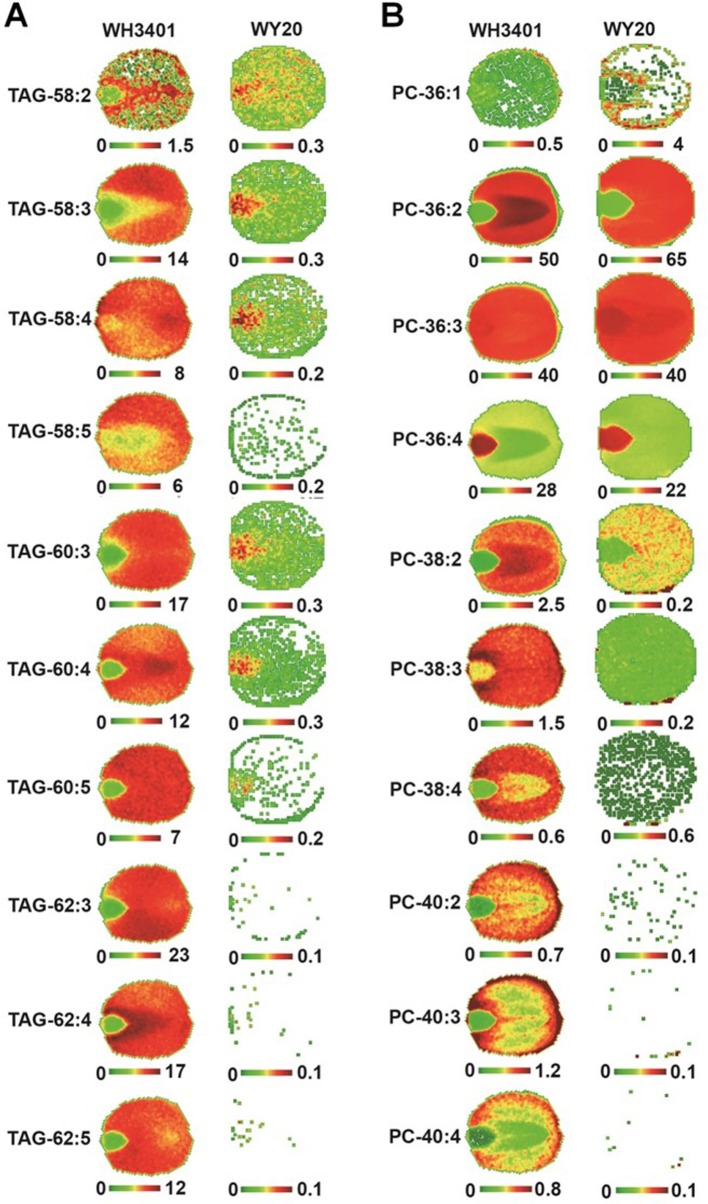
Spatial distribution of triacylglycerol (TAG) and phosphatidylcholine (PC) species in mature seeds. False-colored images of embryo cross sections showing the spatial distribution of selected TAG **(A)** and PC **(B)** species in WH3401 and WY20 seeds. Three biological replicates with consistent results have been analyzed (**Figure S3**).

**Figure 4:**
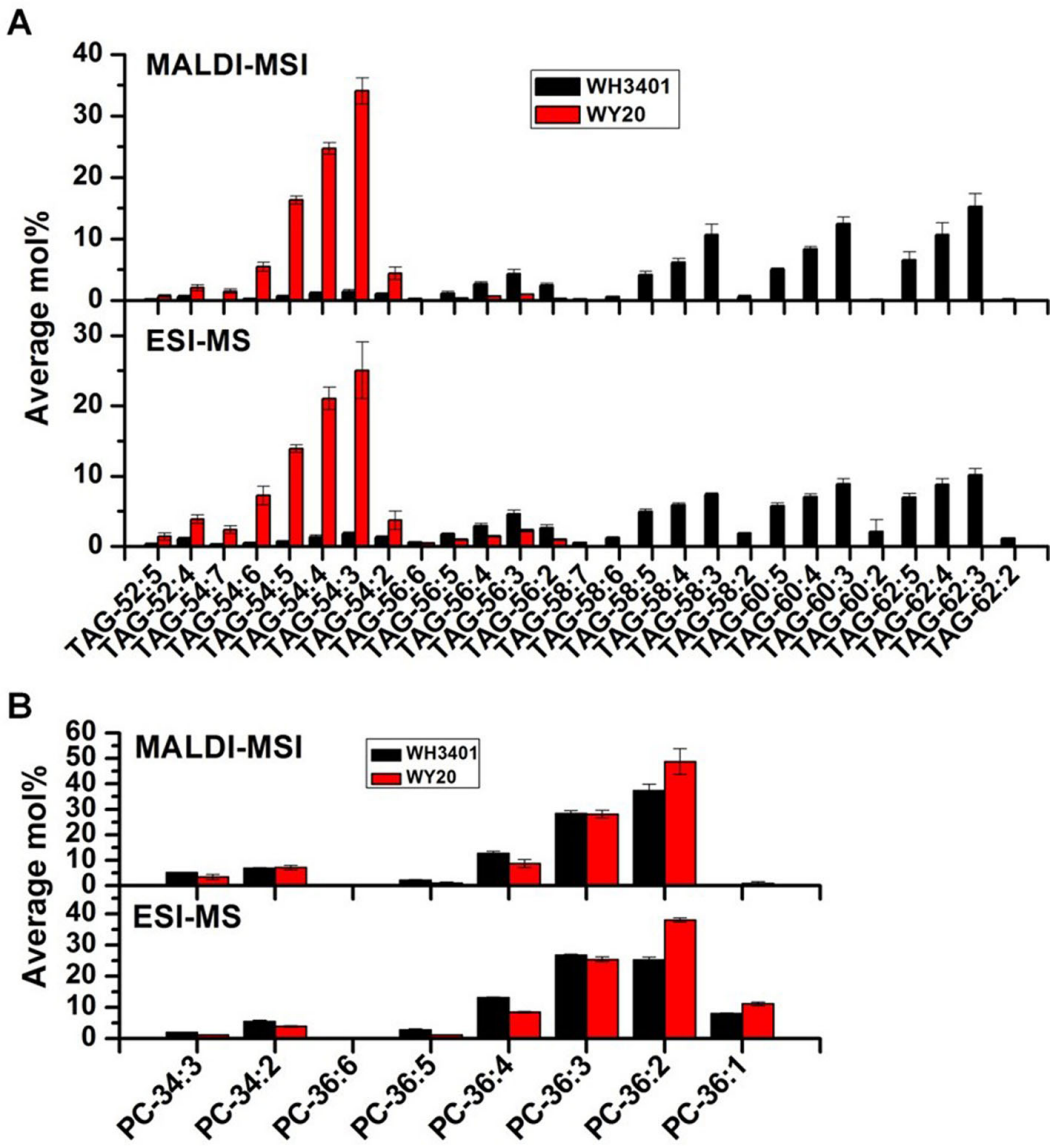
Comparison of the relative levels of triacylglycerol (TAG) and phosphatidylcholine (PC) species in mature seeds measured by MADLI-MSI (mol% from ion intensities summed over the entire section) and ESI-MS of whole-seed extracts using tri-17:0 TAG and di-14:0 PC as a quantitative standard. **(A)** TAG species. **(B)** PC species. Data are the means ± SD of 3 biological replicates.

For TAGs, the 50C and 52C series had similar distribution pattern between both accessions, and heterogeneity was mainly observed between the cotyledonary tissues and embryonic axis, where these species were preferentially localized to the embryonic axis (**Figure S1**). Since the 18C fatty acids (especially C18:1) were the major fatty acids in the low-erucic accession (WY20), the corresponding 54C TAGs, mainly TAG-54:3, TAG-54:4, and TAG-54:5, were the most abundant TAG species in this accession (**Figure 4A**), and they were either more enriched in the cotyledons (e.g. TAG-54:3) or evenly distributed throughout the seed tissues (e.g. TAG-54:4 and TAG-54:5) (**Figure S1**). The other, minor 54C TAGs in WY20 were mainly localized in the embryonic axis (**Figure S1**). Conversely, in WH3401 (high-erucic), the 54C TAGs were significantly less abundant (**Figure 4A**), and were almost exclusively localized to the embryonic axis (**Figure S1**). Another major difference between both accessions is in the relative abundance of the high-molecular weight TAGs such as the 58C, 60C, and 62C series. These TAGs were the most abundant TAG molecular species in the high-erucic accession (**Figure 4A**), which is consistent with the high abundance of the C20:1 and C22:1 fatty acids in this accession, while they were barely detectable in the low-erucic accession that lacks the C20:1 and C22:1 fatty acids (**Figures 3A** and **4A**). In the high-erucic accession (WH3401), these high-molecular weight TAGs were more enriched in the cotyledonary tissues (**Figure 3A**).

For PCs, the 34C PC species were mainly localized to the embryonic axis in both accessions, except for PC34:1 which was localized to both cotyledons and the embryonic axis (**Figure S2**). The 36C PCs, mainly PC-36:2, PC-36:3, and PC-36:4, were the major PC species in both accessions (**Figure 4B**). These molecular species also had similar distribution patterns in both WY20 and WH3401, where PC-36:2 (the most abundant PC) was localized to the cotyledons, while PC-36:4 had a preferential localization in the embryonic axis, and PC-36:3 was uniformly distributed throughout the seed tissues (**Figure 3B**). The main difference in PC distribution between both accessions is the presence of 38C and 40C species in the high-erucic seeds, which were more enriched in the cotyledonary tissues, while these molecular species were absent in the low-erucic seeds (**Figure 3**). However, based on overall mol%, these very long chain PCs (38C and 40C) represented a relatively minor contribution to the PC pool in the high-erucic accession (WH3401; e.g., see scales are mostly less than 2 mol%). The observed distribution patterns of TAG and PC molecular species were consistent in three different biological replicates (**Figure S3**).

### Gene Expression in Seed Tissues of the High- and Low-Erucic Accessions

**Figure 5:**
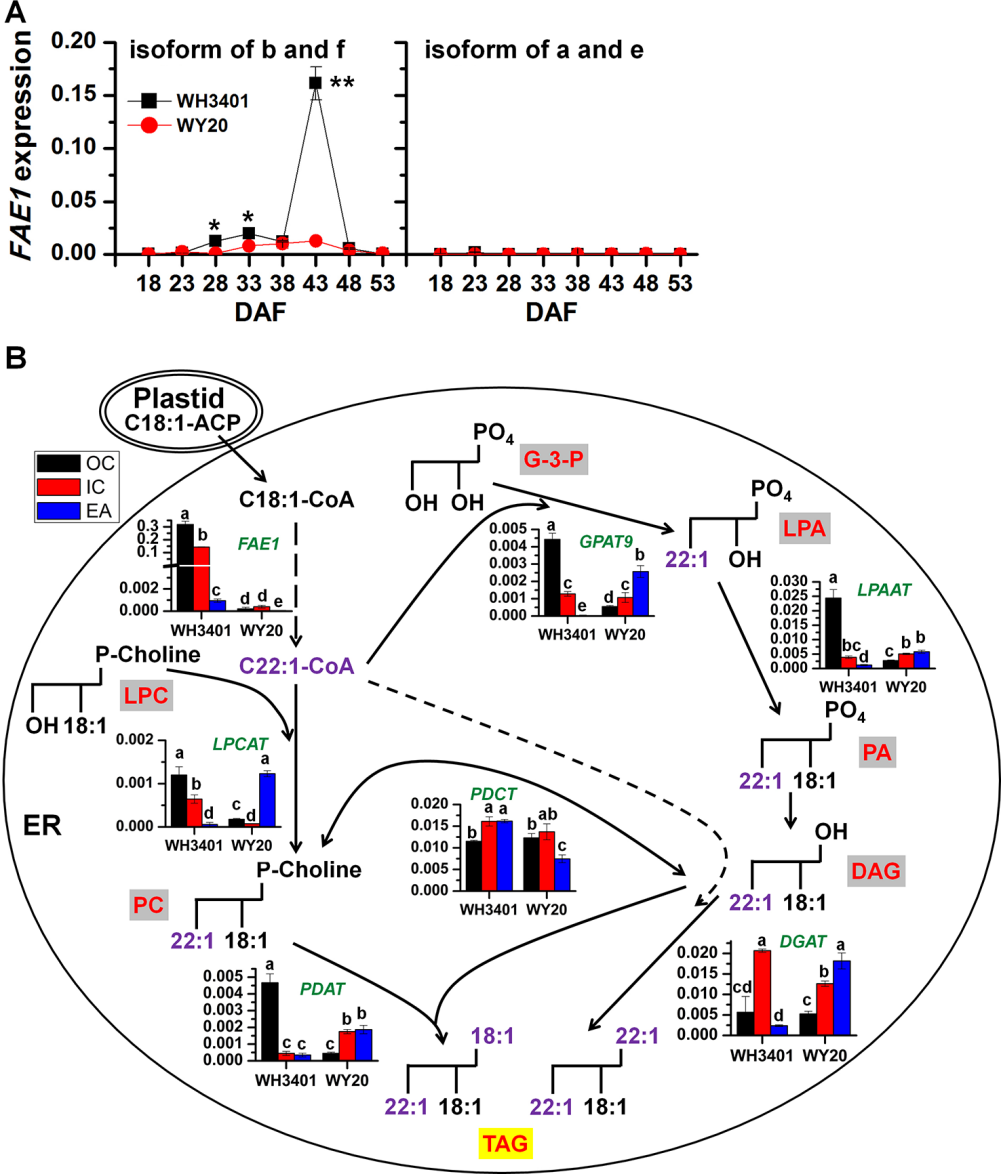
The gene expression change of *FAE1s* in developing seeds and a model of TAG accumulation explaining C22:1 flux into TAG (means ± SD, n = 3). **(A)** Total expression level of the b and f isoforms, as well as the a and e isoforms of *FAE1* in the whole seeds during seed development. * and ** denote significant difference at *P* < 0.05 and *P* < 0.01, respectively, based on Student's *t* test. **(B)** Gene expression levels in the different tissues of 43-day-old seeds mapped to the TAG biosynthesis pathway. Data represent the average of gene expression level of 3 biological replicates. Letters denote significant difference at *P* < 0.05 using ANOVA analysis. Green italic letter denotes gene names, while red letter with gray or yellow background frame denotes lipid classes. EA, embryonic axis; IC, inner cotyledon; OC, outer cotyledon; G-3-P, glycerol-3-phosphate; GPAT9, glycerol-3-phosphate acyltransferase 9; LPA, lysophosphatidic acid; LPAAT, lysophosphatidic acid acyltransferase; PA, phosphatidic acid; DAG, diacylglycerol; DGAT, diacylglycerol acyltransferase; TAG, triacylglycerol; FAE1, fatty acid elongase 1; LPC, lysophosphatidylcholine; LPCAT, lysophosphatidylcholine acyltransferase; PC, phosphatidylcholine; PDCT, phosphatidylcholine:diacylglycerol cholinephosphotransferase; PDAT, phospholipid:diacylglycerol acyltransferase.

Phylogenetic analysis of *FAE1* in *B. napus* revealed that there are 6 different *FAE1* genes (Chalhoub et al., 2014), denoted as *FAE1a-f* (**Figure S4A**). Analysis of the amino acid sequences of the 6 isoforms of FAE1 protein indicated that both FAE1a and FAE1e, as well as FAE1b and FAE1f, are more closely related to each other (**Figure S4B**). Analysis of *FAE1* expression in whole-seeds of both accessions during development showed that only the b and f isoforms were expressed in WH3401 seeds, with the highest expression detected at 43 DAF, while no *FAE1* expression was detected in WY20 at any of the selected time points (**Figure 5A**). Therefore, 43 DAF time point was selected to perform comprehensive transcriptomic analysis in dissected seed tissues of both accessions (**Figure 5B**).

FAE1 expression in the dissected seed tissues showed that *FAE1* was highly expressed in the outer and inner cotyledons of WH3401, relative to the EA, and as expected, it was barely expressed in all tissues of WY20 (**Figure 5B**). The expression pattern of *FAE1* in WH3401 was consistent with the fatty acid composition analysis showing that erucic acid (C22:1) was the most abundant FA species in the outer and inner cotyledons. It is also consistent with the MALDI-MSI results, where the erucic acid-containing TAGs (e.g. the 58C, 60C, and 62C series) were more enriched in the cotyledonary tissues than the EA.

The expression levels of the different genes involved in TAG biosynthesis in the ER were compared among the different seed tissues of the high- and low-erucic accessions (**Figure 5B**). There are two possible routes that can lead to TAG assembly in the ER. One route is through the conventional Kennedy pathway, which utilizes glycerol-3-phosphate as initial substrate and includes four sequential enzymes, glycerol-3-phosphate acyltransferase 9 (GPAT9), 1-acylglycerol-3-phosphate acyltransferase (LPAAT), phosphatidic acid phosphatase (PAP) and diacylglycerol acyltransferase (DGAT) (Chapman and Ohlrogge, 2012). In WH3401, *GPAT9* had the highest expression in the OC, while in WY20, it was relatively more expressed in the EA than the cotyledonary tissues. For *LPAAT*, it was highly expressed in the OC of WH3401, relative to all the other tissues of both accessions. In WH3401, *DGAT* was expressed to higher levels in the IC than the other two tissues, while in WY20, it was expressed in EA > IC > OC (**Figure 5B**). The other pathway of TAG assembly involves the transfer of an acyl chain from the acyl-CoA pool to lysophosphatidylcholine (LPC) to form PC, and then from PC to diacylglycerol (DAG) to form TAG, *via* the action of two enzymes, lysophosphatidylcholine acyltransferase (LPCAT) and phospholipid:diacylglycerol acyltransferase (PDAT), respectively (Chapman and Ohlrogge, 2012). In WH3401, *LPCAT* was more expressed in the cotyledonary tissue than the EA, while opposite pattern was observed in WY20. *PDAT* had a similar expression pattern to that of *LPAAT*, where the highest expression level was observed in the OC of WH3401 compared to all the other tissues of both accessions (**Figure 5**). Based on these results, it seems that both pathways could be contributing to the assembly of erucic acid-containing TAGs in WH3401, since the expression pattern of almost all the genes of both the TAG biosynthesis pathways suggests a more enrichment in the cotyledonary tissues, relative to the EA, which is consistent with the observed cotyledonary localization of erucic acid-containing TAGs in this accession. However, the affinity of native lysophosphatidic acid acyltransferase (LPAAT) is poor for fatty acyl chains with more than 18 carbons, implying C22:1 is difficult to incorporate into the *sn-2* position of lipids by the Kennedy pathway (Lassner et al., 1996; Furmanek et al., 2014; Kaur et al., 2019) (**Figure 5**). This may suggest that LPCAT and PDAT help to compensate for this deficiency of LPAAT by introducing erucic acid into the *sn*-2 position of TAGs. Further positional analysis of PC and glycerolipid molecular species will be necessary to confirm this speculation. In any case, it seems that there is a complex and heterogeneous distribution of TAG pathways that contributes to the enrichment of erucic acid in TAG in cotyledons.

## Conclusion

Here, the distribution of the erucic acid in *B. napus* seeds, and the transcripts encoding the elongation enzymes primarily responsible for its synthesis, were identified. As expected, the distribution of the *FAE1* transcripts were associated with the distributions of total erucic acid in seed parts analyzed by GC-FID, or in TAGs analyzed by ESI-MS (in extracts) or by MALDI-MSI (*in situ* in seed sections). The pathways for TAG assembly of erucic acid-containing TAGs could be through either DGAT or PDAT based on expression patterns and pathway analysis. Overall, these studies shed light on the spatial complexity of TAG assembly in *B. napus* seeds, especially for erucic acid-containing TAGs.

## Data Availability Statement

The datasets generated for this study are available on request to the corresponding authors.

## Author Contributions

LG, KC, and SL designed and supervised the study. SL, MA, and DS performed the experiments and data analysis. SL and MA prepared the manuscript. LG, KC, and DS revised the manuscript. All the authors read and approved the manuscript.

## Funding

This work was supported by National Natural Science Foundation of China (31701458, 31871658) and a grant from the U.S. Department of Energy, Biological and Environmental Research (BER) program under contract # DE-SC0020325.

## Conflict of Interest

The authors declare that the research was conducted in the absence of any commercial or financial relationships that could be construed as a potential conflict of interest.

## Supplementary Material

The Supplementary Material for this article can be found online at: https://www.frontiersin.org/articles/10.3389/fpls.2019.01744/full#supplementary-material
