# Supplementary material for: Heterogeneous Distribution of Erucic Acid in Brassica napus Seeds
Source: Front Plant Sci. 2020 Jan 29;10:1744. doi: 10.3389/fpls.2019.01744 (PMC7001127; doi:10.3389/fpls.2019.01744)
Supplement: Supplementary file 1 [file Presentation_1.pptx]

## Slide 1
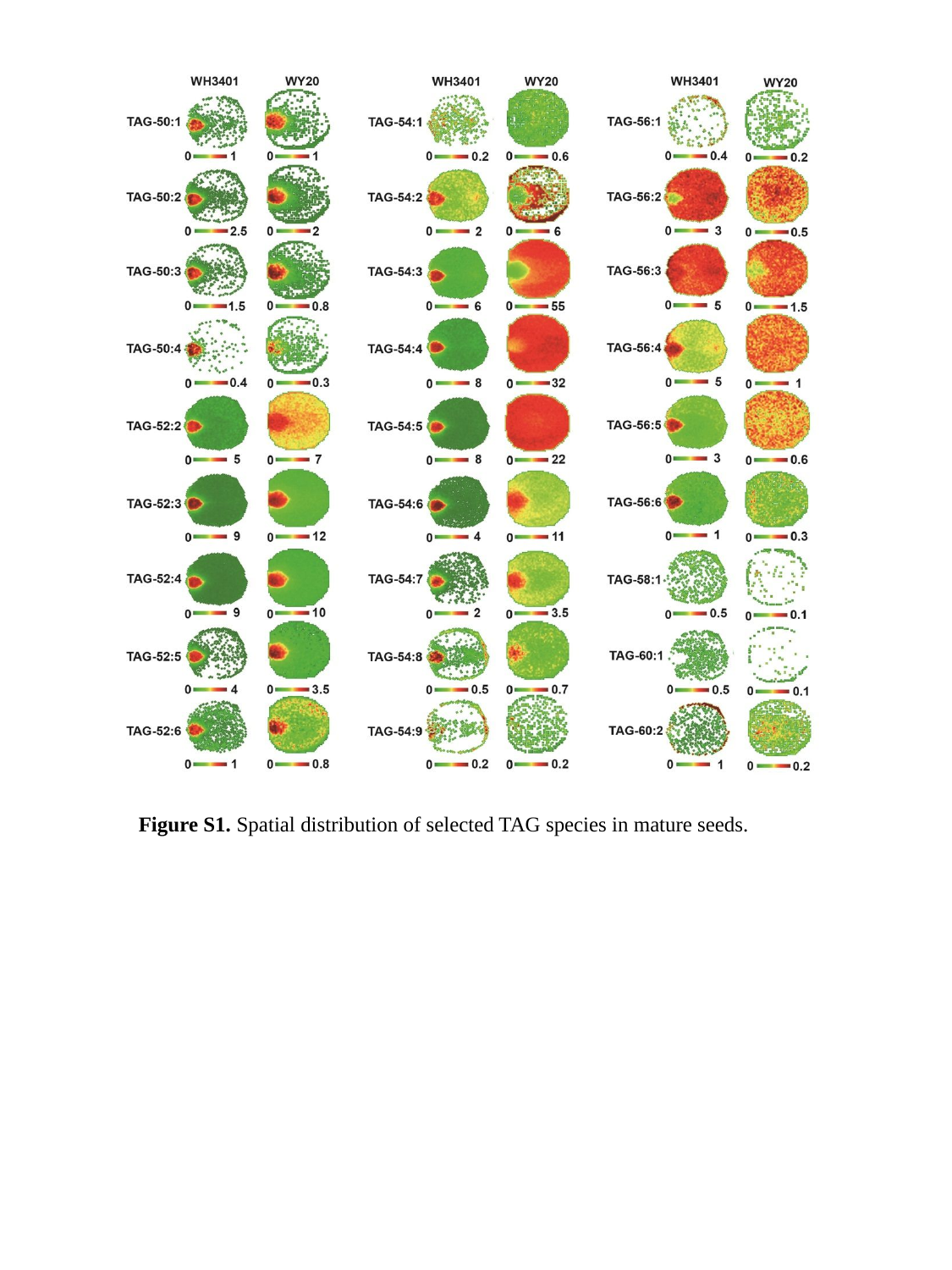

Figure S1. Spatial distribution of selected TAG species in mature seeds.

## Slide 2
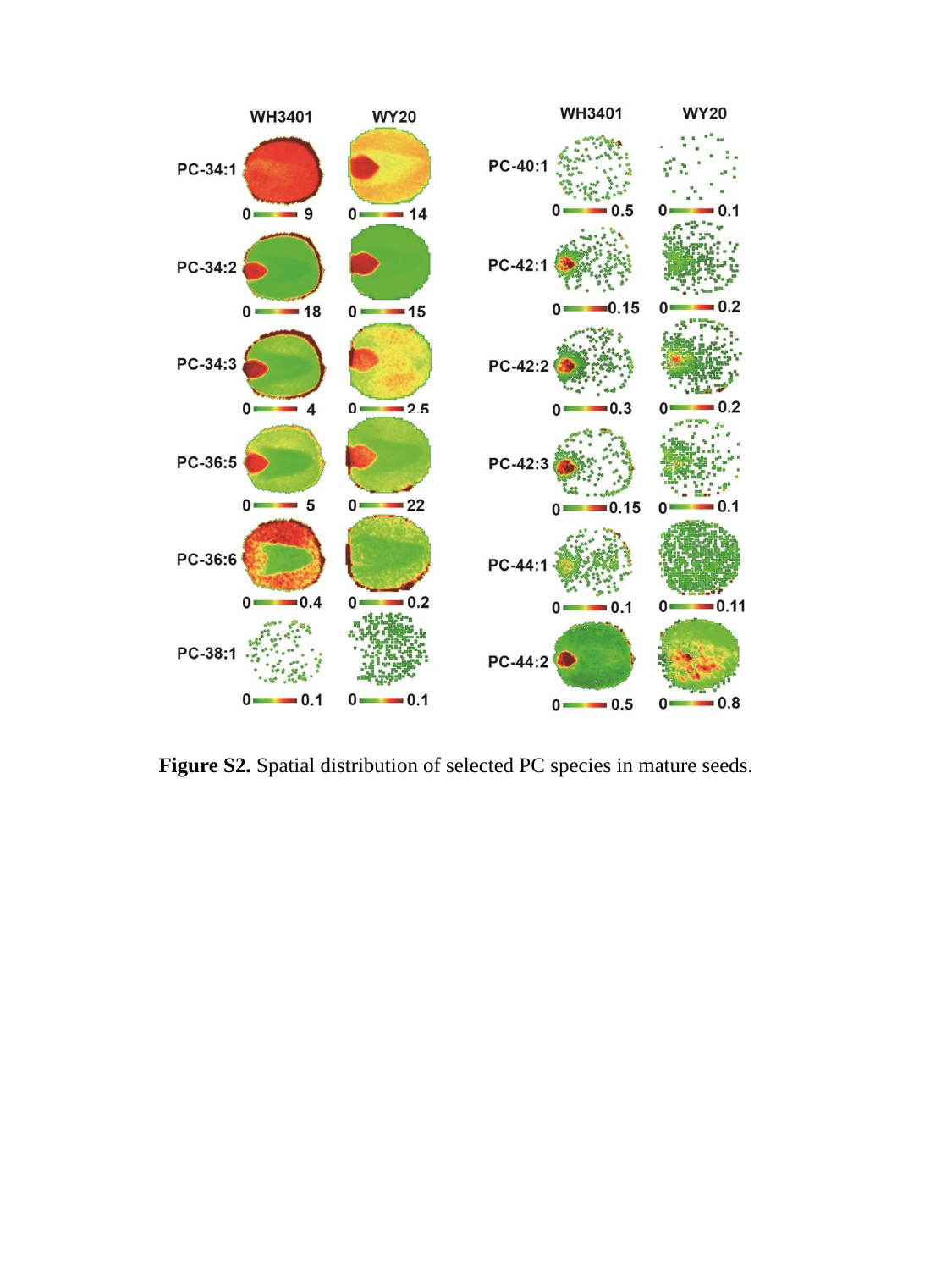

Figure S2. Spatial distribution of selected PC species in mature seeds.

## Slide 3
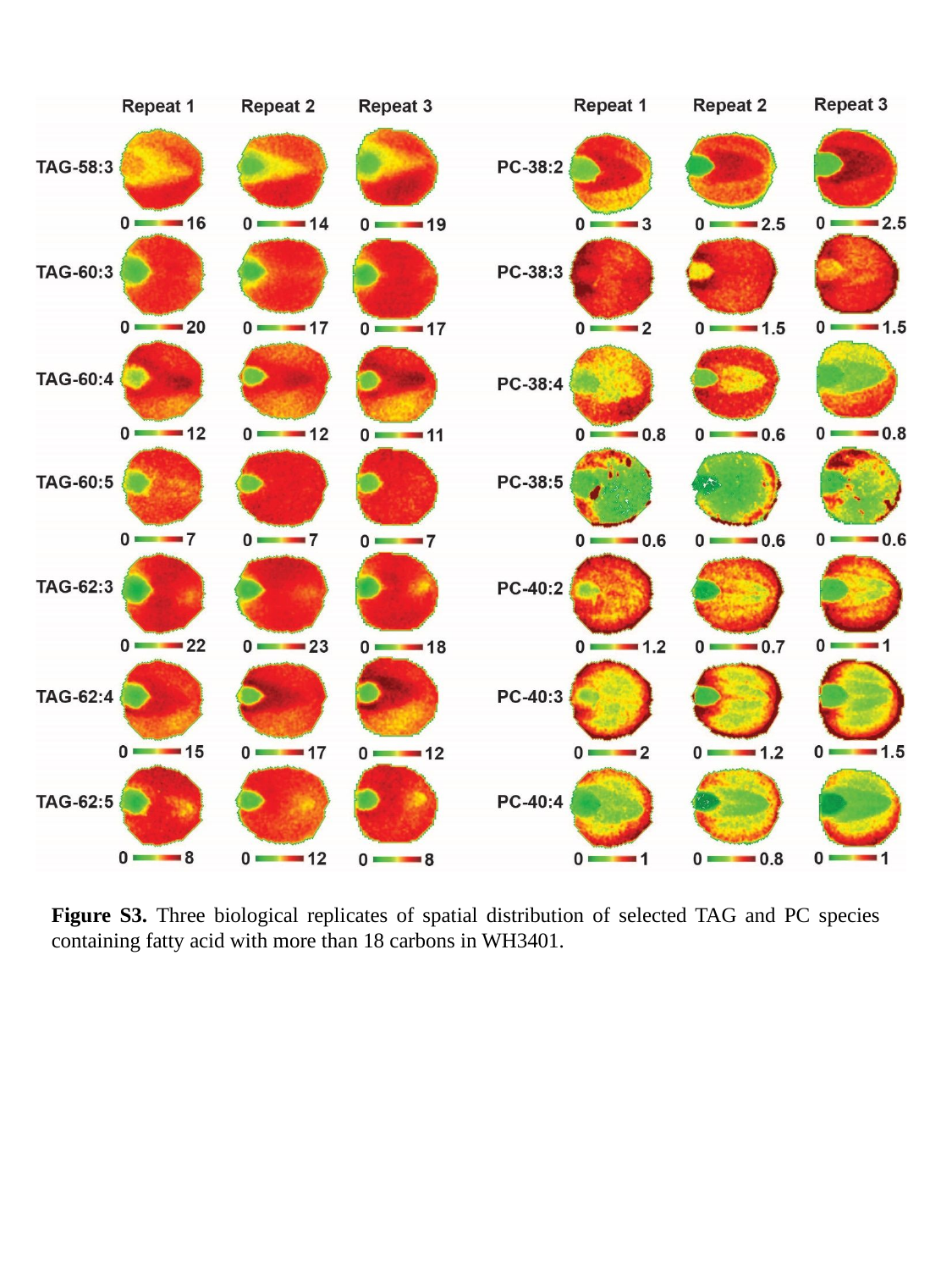

Figure S3. Three biological replicates of spatial distribution of selected TAG and PC species containing fatty acid with more than 18 carbons in WH3401.

## Slide 4
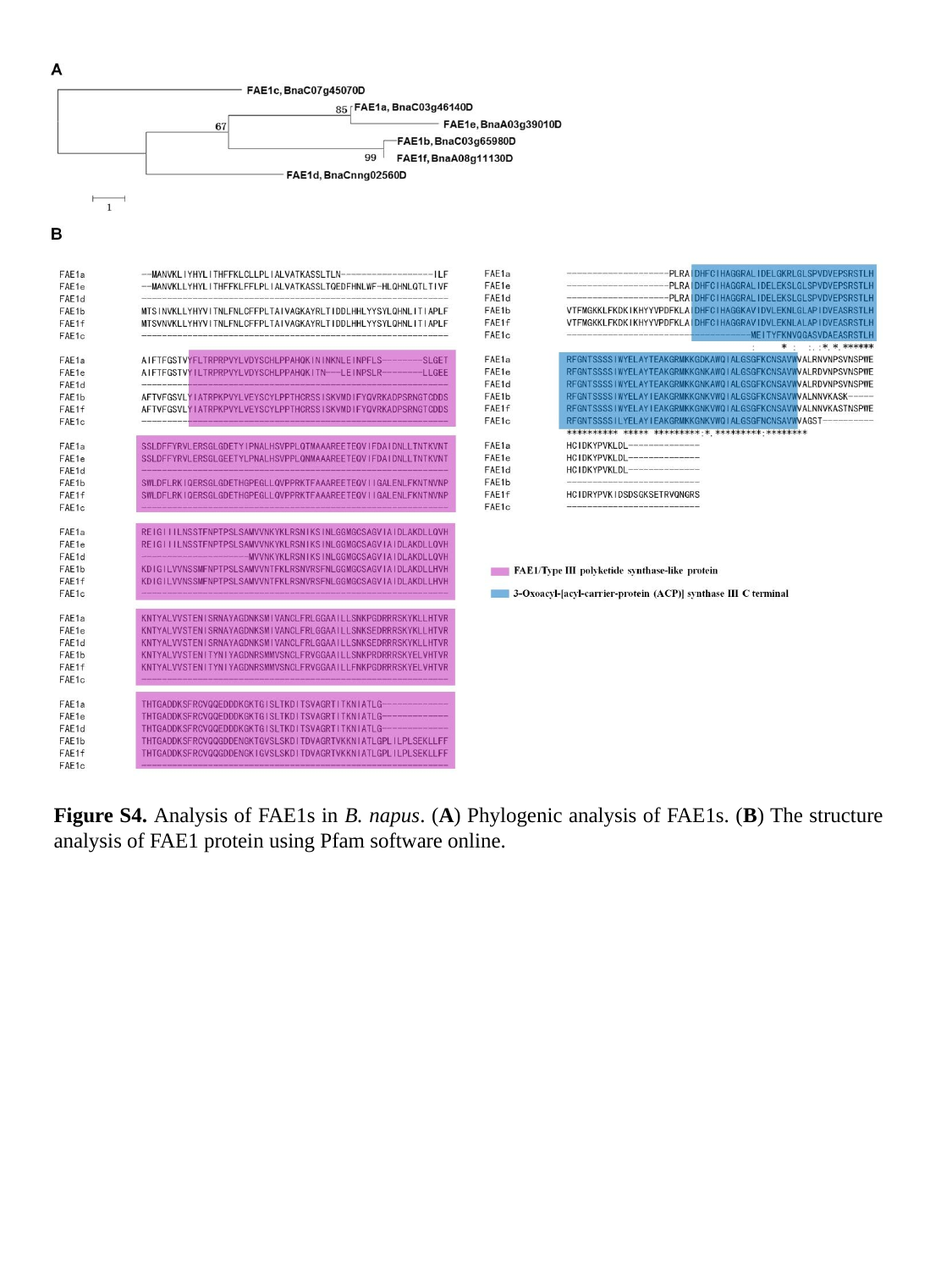

Figure S4. Analysis of FAE1s in B. napus. (A) Phylogenic analysis of FAE1s. (B) The structure analysis of FAE1 protein using Pfam software online.

## Slide 5
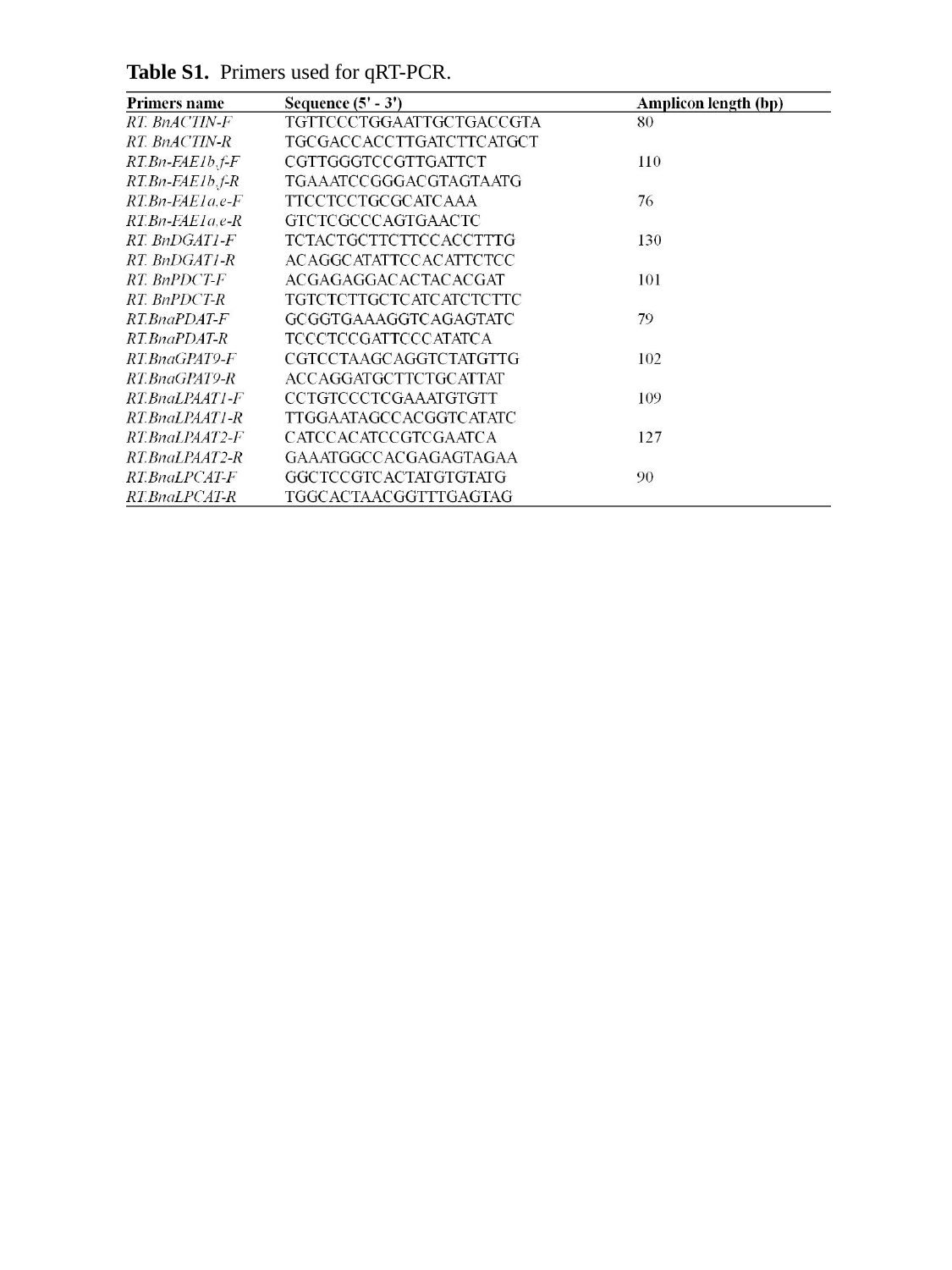

Table S1. Primers used for qRT-PCR.
